# Natural anti-CCR5 antibodies in HIV-infection and -exposure

**DOI:** 10.1186/1479-5876-9-S1-S4

**Published:** 2011-01-27

**Authors:** Lucia Lopalco

**Affiliations:** 1 Division of Immunology, Transplantation and Infectious Diseases, San Raffaele Scientific Institute, Milan, Italy

## Abstract

Natural antibodies constitute a first-line of defence against pathogens; they may also play other roles in immune regulation and homeostasis, through their ability to bind host antigens, surface molecules and receptors. Natural anti-CCR5 antibodies can be decisive in preventing HIV infection in mucosal tissues and offer prompt and effective protection just at major sites of virus entry. Among natural anti-CCR5 antibodies, IgG and IgA to the ECL1 domain have been shown to block HIV effectively and durably without causing harm to the host. Their biological properties and their uncommon generation in subsets of HIV-infected and HIV-exposed individuals (so called ESN) will be introduced and discussed, with the aim at exploiting their potential in therapy and prevention.

## Natural antibodies

Human serum usually contains natural IgG, IgM and IgA antibodies, generated independently of any exposure to foreign antigens or vaccines or elicited in the course of infectious or autoimmune diseases. Most of these natural antibodies also are polyreactive, i.e. able to bind various antigens; they are often self-reactive, i.e. capable of recognizing some host antigens. Natural antibodies are generated by the B-1 subset of B cells without the intervention of T cells, therefore belong to the innate arm of the immune system [1].

B-1 cells are found in peritoneal and pleural cavities where they provide first-line defence through antibodies able to bind polysaccharide antigens and repeated motifs that are typically found in microbial cell walls and macromolecules [2,3].

Innate defences are important in cutaneous and especially in mucosal linings, that are the host physical boundaries with the environment; here, natural, polyreactive IgM and IgA antibodies, produced by the “primordial”, T-independent B cells, control auto-antigens, exogenous antigens and microbes. Specific, monoreactive antibodies from the adaptive B-cell system (the large, B-2 subset) are produced later, after the activation and recruitment of T-cells. In other words, if the antigen-antibody reaction is compared to a “key-and-lock” model, natural antibodies found in human secretions act as “passe-partout” keys to offer a background protection against most pathogens, food antigens and microbes, before the antigen-specific response can develop [4]. B-1 cells features and activities are still largely unknown, especially in human immunology, and are currently an active field of investigation. According with studies of cell transplants performed in transgenic mice, B-1 population can be divided in two further subset (B-1a and B-1b), which display different phenotypes, origins and functions. CD5^+^ B-1a cells stem from fetal cells and can self-replicate, while CD5^─^ B-1b cells derive from bone marrow precursors common to B-2 cells, that constitute the large majority of the B cell population [2]; however, recent experiments have observed the development of both B-1 cell subtypes from bone marrow cell lineages [5,6]. Most B-1 cells display a reduced BCR diversity and affinity, due to the lack of somatic recombination and to the poor activity of receptor editing, that increases with age [6,7]. Most natural antibodies are IgM [8], but B-1 cells undergoing immunoglobulin class switch have been recently described [9].

B-1a cells become activated in response to antigens stimulation [10], and can directly produce antibodies without the intervention of T-helper cells, while B-1b cells can take part in adaptive immunity by providing a specialized type of IgM memory cells [11-13].

Several functions have been proposed for natural antibodies, including a first–line role in the defense against infections, a scavenger-like activity to apoptosis by-products and a turn-off, regulative role in the maintenance of immune homeostasis [7,14]. Not surprisingly, pools of intravenous immunoglobulins from healthy donors were shown to contain antibodies directed against several cell surface molecules, including CD4, CD5, cytokine receptors, adhesion motifs and CD95 (Fas receptor) [15].

Natural, polyreactive and anti-self antibodies have been also found in mucosal secretions, such as colostrum and saliva [1,4,16]; high-specific S-IgA were observed in mucosal secretions, where showed a stronger anti-bacterial activity than their serum counterparts, supporting the primary role of S-IgA in controlling mucosal infections.

B-1 cells are more prompt than B-2 subpopulation to switch to IgA production in response to antigen stimulation; their contribution accounts for half of IgA found in serum or in intestinal lamina propria [17]. Antimicrobial efficiency of S-IgA was found to be enhanced by their binding to pFv, a gut-associated molecule, suggesting that these immunoglobulins took part in controlling gut infections [4]. How natural antibodies can bind unrelated epitopes, instead of exhibiting the conventional monoreactivity, is still undetermined; some studies suggested a role for the CDR3 framework region of the heavy immunoglobulin chain, a domain where even single mutations might dramatically alter the specificity and/or the affinity of the antibody molecule for its target antigen [18,19].

Natural reactive autoantibodies recognizing CCR5 have been isolated from pools of immunoglobulins from healthy donors [15]. The role of similar responses, generated in the absence of autoimmune diseases, is still debated; antibodies to CCR5 and to other immune receptors and mediators were supposed to be involved in the maintenance of immune homeostasis. As an example, anti-CCR5 antibodies could limit the migration of CCR5+ proinflammatory cells (e.g. macrophages, dendritic cells, CTLs and Th1 lymphocytes) toward inflammatory sites releasing CCL5/RANTES, CCL3/MIP-1alpha or CCL4/MIP-1beta, in order to limit excessive and harmful effects of inflammation [20]. More importantly, anti-CCR5 natural antibodies also showed HIV-blocking properties [15,21,22]. Anti-CCR5 antibodies were even found in CCR5-lacking subjects, homozygous carriers of the Delta32 mutation, after repeated exposure to partner’s CCR5+ cells through sexual activity [21]. The finding is not surprising, since allogeneic immunization has been shown to induce anti-CCR5 antibodies [23].

Different types of antibodies to CCR5 have been isolated from HIV-infected and from HIV-exposed, seronegative (ESN) subjects. Most anti-CCR5 antibodies recognized the N-terminus and especially the second extracellular loop (ECL2) of the receptor, the immunodominant region involved in chemokine and in HIV binding [1,15,16,21,24,25]. According to studies employing anti-CCR5 monoclonal antibodies (mAbs), some of the immunoglobulins with these two specificities competed for chemokine binding, blocked HIV docking or, more significantly, prevented cell fusion and virus entry [26,27].

A special subset of anti-CCR5 antibodies recognized the first external loop of CCR5 receptor (ECL1), a domain not involved in ligand binding or in HIV docking. Anti-CCR5 antibodies to the ECL1 domain have been detected in serum and mucosal secretions from exposed, HIV-negative (ESN) people and in some HIV-positive subjects, both men and women. A study observed such anti-CCR5 antibodies in thirteen out of forty-six HIV-positive donors; the presence of anti-CCR5 antibodies was not significantly associated with either CD4+ T-cell counts (range: 19-1,047 cells/mL) or with viral load (range: <50–119,000 copies/mL) [28]. However, findings from broader cohorts, including subpopulations of HIV progressors and long-term non progressing (LTNP) individuals, support the hypothesis that anti-ECL1 IgG and IgA may be involved in HIV protection or in the infection control [22,29,30].

## Anti-CCR5 antibodies in mucosal secretions

Anti-CCR5 antibodies were isolated not only from the sera of HIV-infected or ESN individuals but also from all types of mucosal secretions, such as saliva, breast milk and genital secretions [1,16,29,30].

Anti-CCR5 antibodies were isolated from serum and mucosal secretions of eight out of 118 ESN individuals, all sexual partners of HIV-positive patients: interestingly, all males and females carrying CCR5-specific genital IgA also displayed salivary antibodies, a finding showing the generation of both systemic and local responses to HIV exposure. Anti-CCR5 antibodies specifically recognized synthetic peptides corresponding to ECL2 and ECL1 domains and blocked HIV infectivity in PBMC cells [29].

IgG and IgA specific to the ECL2 domain of CCR5 receptor were found in breast milk of HIV-negative and HIV-positive women (66% and 83%, respectively). Despite the higher avidity shown by antibody pools from HIV-positive women, both anti-CCR5 antibodies inhibited up to 75% infection of macrophages and dendritic cells with HIV isolates, showing that natural antibodies provided by breast milk could protect newborns from HIV transmission [16]. Further results supporting a possible role of natural anti-CCR5 antibodies also came from the study of an African cohort of newborns from HIV-positive mothers. Twenty-five out of thirty-three children remained uninfected and showed high levels of specific anti-HIV neutralizing antibodies [31]; a subset of these children and their mothers also displayed HIV-blocking antibodies to CCR5 [32].

Cervicovaginal secretions contain large amounts of polyreactive natural antibodies, mainly IgG and S-IgA and, to a lesser extent, IgM; in these pools, anti-CCR5 immunoglobulins have been found by different investigators [1,24,29,33]. Natural anti-CCR5 antibodies from cervicovaginal fluid samples isolated from HIV-negative women reduced levels of infection in macrophages and dendritic cells challenged with R5- but not X4- HIV strains [1]. Genital and salivary IgA to the ECL1 domain specifically competed with chemokines for CCR5 binding and downregulated the receptor from PBMC surface; more importantly, only natural antibodies with this specificity blocked HIV transcytosis across an epithelial cell layer mimicking the human mucosa. This result was noteworthy, because other anti-CCR5 antibodies, such as 2D7, that prevented HIV interaction with CCR5 coreceptor by binding to ECL2 domain, was unable to block virus transcytosis [22,33,34].

A broad clinical study searched for anti-CCR5 antibodies to ECL1 in 497 subjects, including 85 LTNPs, 70 HIV-progressors, 135 HIV-positive patients receiving highly active antiretroviral therapy (HAART) and 207 HIV-negative donors [30]. Anti-CCR5 antibodies were isolated in 23% of the LTNP subjects but not in the other subpopulations studied (*P*<0.001). Anti-CCR5 antibodies recognized a conformational epitope within the ECL1 domain and induced a stable and long-lasting downregulation of CCR5 from the surface of T lymphocytes, thereby inhibiting HIV entry. Receptor internalization was specifically inhibited by sucrose, but not by filipin or nystatin, nocodazole or cytochalasin D, thus supporting a specific role for clathrin-coated pits and excluding the caveolae compartments [30]. In addition, CD4^+^ lymphocytes from the LTNP subpopulation who displayed anti-CCR5 antibodies were resistant to *in vitro* infection with R5-tropic HIV-1 strains, due to CCR5 downregulation; finally, anti-CCR5 antibodies blocked *in vitro* infectivity of HIV primary isolates belonging to clades A, B and C. The level of ECL1-specific anti-CCR5 antibodies appeared to be correlated with levels of HIV exposure, being lower in seronegative ESN subjects and higher in seropositive LTNP individuals (0.1% *vs*. 8% of the total antibodies, respectively).

Interestingly, the loss of anti-CCR5 antibodies was observed in the course of the clinical follow-up and this event was significantly associated with clinical progression toward disease in 9 out of 20 LTNP enrolled in the study; these LTNPs experienced a significant increase in viremia and required therapy, thus becoming “progressors”. Strikingly, patients who retained anti-CCR5 antibodies did maintain a stable LTNP status without any treatment. According to the finding, the loss of anti-CCR5 Abs was associated with progression toward disease; this observation was strongly supported by the development of AIDS in some patients despite antiretroviral therapy [30].

## CCR5 domains and HIV binding: lessons from anti-CCR5 mAbs

**Figure 1:**
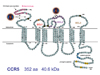
**CCR5 coreceptor.** Scheme illustrating the three-dimension structure of CCR5 coreceptor. Extracellular domains show the HIV binding sites and the immunodominant epitopes mapped by mAbs and by natural anti-CCR5 antibodies. Sites of O-Glycosylation (Ser6), palmitoylation (Cys321, 323 and 324) and phosphorylation (Ser336, 337, 342 and 349) are also shown. C20-C269 disulphide bond is represented in an open form.

Several studies employing monoclonal antibodies have defined CCR5 epitopes involved in major receptor functions, such as binding to chemokines, activation, trafficking and HIV docking. Some of these antibodies, such as MC-1 or PA14, were found suitable to work as therapeutic inhibitors of viral entry, due to their ability in inhibiting gp120 binding or in promoting CCR5 internalization without triggering intracellular signaling; the humanized version of PA14, PRO140, has been tested in clinical studies [26,27,35]. A scheme representing CCR5 molecule, its binding domains and the key epitopes mapped on its structure is illustrated in **Figure **1. Similarly to other G-protein coupled receptors (GPCRs) and membrane-associated proteins, CCR5 is poorly immunogenic; its four extracellular domains represent about one fourth of its whole sequence (90 out of 352 aminoacids); the two longer domains, the N-terminus and the second extracellular loop (ECL2), span about 30 aminoacids each [36]. These latter domains host immunodominant epitopes recognized by the majority of monoclonal antibodies, such as D2-Y3, Y10-D11 or K171-E172 [36]; both the N-terminus and the ECL2 domain are also involved in chemokine and HIV binding [26,36,37].

Alanine mapping and point mutation studies have identified critical aminoacids on the CCR5 molecule, leading to design epitope maps and theoretical models representing the extracellular domains of the receptor and their hypothetical interactions [26,27]. Not surprisingly, few monoclonal antibodies were able to bind native and denatured CCR5 in Western blot assays, a finding showing that most CCR5 epitopes are conformation-sensitive [37]. Key aminoacids included in discontinuous, conformational epitopes may embrace one or more residues among the first 20 aminoacids in the N-terminus, other aminoacids in two distinct regions within the ECL2 domain and single aminoacids belonging to other domains, such as the D95 residue located in the ECL1 [36]. 2D7, one of the most potent antibodies described in many studies, binds to aminoacids Q170-K171-E172 and W190 in the ECL2 domain [26,27,37]. Antibodies targeting the N-terminus domain of CCR5, as MC-5 or PA9, competed for binding of soluble gp120-CD4 complex with high affinity, but were less effective than the ECL2-specific antibodies in preventing cell-cell fusion and virus entry [27,38]. Conversely, antibodies to the ECL2 domain, as 2D7, did not prevent gp120-CD4 complex binding effectively but were strong inhibitors of HIV entry; these findings supported a model of dual interaction between CCR5 and HIV, where the first interaction, involving the binding between V3 stem on the viral protein and the N-terminus of the coreceptor, occurred before the second one, which took V3 crown in close contact with the ECL2 domain and triggered HIV envelope-cell membrane fusion; both interactions with N-terminus and ECL2 domains were required for HIV docking [27,36].

The ECL2 domain hosts both HIV- and chemokine-specific binding sites; antibodies recognizing this domain were effective in preventing chemokine binding and/or signaling [37]. Antibodies recognizing conformational epitopes spanning different extracellular domains of the CCR5 molecule displayed different ability in inducing ligand binding, signaling and receptor trafficking (e.g. desensitization, phosphorylation, downregulation). For example, the MC-6 antibody activated CCR5 but was unable to induce receptor internalization, whereas MC-1 caused CCR5 internalization, via cholesterol-rich raft domains; MC-4 specifically inhibited CCL5/RANTES-mediated endocytosis, but did not affect chemokine signaling [26]. PA9 and PA12, all recognizing CCR5 N-terminus, were ineffective at blocking intracellular signaling, while PA14 and 2D7 prevented intracellular calcium mobilization induced by chemokine binding [27]. The wide spectrum of effects mediated by the binding of different antibodies supported the existence of multiple conformations for CCR5 molecules [26]. Most importantly, the modulation of specific events associated with the coreceptor, such as ligand binding, signaling and downregulation, opened the way to the use of monoclonal antibodies as therapeutic tools, capable of preventing HIV spread by steric hindrance and/or by receptor internalization without affecting physiologic chemokine signaling. Moreover, antiviral antibodies could also fight viruses by inducing antibody-dependent cellular cytotoxicity (ADCC), by virus opsonization and by recruiting components of the complement cascade [39]. Another interesting feature shown by some monoclonal antibodies was the possibility to obtain synergistic antiviral activity, due to the existence of various non-overlapping epitopes involved in HIV binding, docking and entry [40].

## The generation of anti–CCR5 antibodies

Anti-CCR5 antibodies have different ways of generation and different mechanisms of action. Anti-CCR5 antibodies to the chemokine or to the HIV binding site, i.e. recognizing the N-terminus and/or the ECL2 domain, usually appear in response to experimental immunization with cells expressing CCR5 or to HIV infection; these antibodies block HIV entry by binding competition or steric hindrance [16,36]. Antibodies to the immunodominant ECL2 domain were also found in Delta32 homozygous subjects, in response to the sexual exposure to CCR5+ cells from their sexual partners; in these cases, CCR5 worked as an alloantigen, similarly to non-self HLA molecules in HLA-discordant sexual partners [21,41,42].

Finally, anti-CCR5 antibodies recognizing the ECL1 domain of the protein can appear in response to HIV exposure, or even independently from it. These antibodies do not interfere with HIV binding directly, but induce co-receptor downregulation, thus blocking virus infectivity by an indirect way [22,34,43].

Anti-CCR5 antibodies to ECL1 domain are not commonly observed in people who are exposed to or become infected with HIV. In order to explain their generation, various hypotheses can be formulated. First, this type of anti-CCR5 antibodies is probably elicited by low levels of HIV-specific stimulation; this idea is supported by the fact that these antibodies have been found in ESN and LTNP people but not in subjects who did not experienced HIV exposure or in fast progressing HIV-positive people. The persistence of very low, undetectable levels of HIV replication may provide a continuous antigen boost that does not result in a strong generalized immune activation; this kind of HIV exposure could be similar to what was observed in the course of natural latent viral infections (*e.g.*, herpesviruses), in the exposure to food-borne antigens and/or to oral vaccines, which may establish tolerance and nevertheless retain their antigenic potential [44,45].

Second, anti-CCR5 Abs can be due to antigenic stimulations other than HIV; this hypothesis finds its root in the natural history of other viral infections, where virus-induced alterations of self antigens can give rise to auto-immunogenic proteins and to the corresponding auto-antibodies [46]. Host factors itself, as endogenous retroviruses (ERVs) or other latent or concomitant viral infections, could induce such perturbations in host cells, finally leading to conformational changes in host receptors and to the reshaping of self proteins in non-self, antigenic epitopes [47-50]. In these rare hosts, CCR5 conformation would be affected by atypical local conditions or by host factors with the potential to induce autoimmune antibodies, that block HIV replication by acting on the coreceptor, rather than on the HIV virus.

Third, some individuals could possess an auto-reactive pool of B-memory cells, due to a previous priming caused by endogenous retroviral proteins sharing homology with HIV env proteins. Once exposed to HIV, these subjects will promptly generate specific responses to antigens mimicking viral particles. The two latter hypotheses found confirmation in the behaviour of some animal ERVs, such as the Jaagsiekte sheep retrovirus (JSRV) or the avian leucosis virus (ALV), which expressed env proteins to prevent infections from exogenous retroviruses [51,52].

In ESN and LTNP individuals who were able to control HIV, host physiological and immunological conditions might have established a positive feedback cycle that maintained undetectable levels of virus replication and a suitable antigen presentation on one hand and long-lasting responses, capable of blocking HIV through its major coreceptor on the other, therefore providing a key mechanism for fighting HIV replication [53]. Another remarkable point in the clinical study previously described was the observation that the viral phenotype in LTNP patients carrying anti-CCR5 antibodies did not change in the presence of such antibodies, thus confirming that the selective pressure of CCR5 inhibitors did not induce a change of viral phenotype *per se*, as already reported in a monkey model [54]. In addition, anti-CCR5 antibodies did not cause any apparent alterations in the immune function, as demonstrated by the continued health status of subjects who retained anti-CCR5 antibodies; both these findings provide an argument against theoretical concerns about CCR5 targeting with specific antibodies.

## Passive immunization to CCR5

Humanized monoclonal antibodies recognizing CCR5 extracellular domains (the N-terminus and/or the ECL2) have been developed and competed with gp120 binding [55]. Passive immunization with humanized monoclonal antibodies may offer several advantages in respect to other antiviral drugs. Monoclonal antibodies are highly target-specific and therefore they minimize side effects or toxicity; their very long plasma half-lives allow biweekly or even monthly administrations; antibodies are proteins administrated intravenously, hence their pharmacokinetics, metabolism and toxicity differ from those of HIV-inhibiting drugs, that are low molecular weight molecules administered per os. Moreover, different anti-CCR5 antibodies can provide different spectra of antiviral and anti-chemotaxis activities. On the other hand, antibody-based drugs also have disadvantages, such as the inconvenience of intravenous administration, the potential for inducing allergic reactions and the possible development of neutralizing anti-antibodies [40].

PRO140 and another antibody, HGS004, have been tested in HIV-infected patients [56,57]. At nanomolar concentrations *in vitro*, PRO140 blocked HIV strains belonging to different clades both in primary macrophages and in PBMC [35]. PRO140 inhibited HIV without blocking the CCR5 response to chemokines, whereas HGS004 prevented both viral infection and chemokine signaling. Notably, antibodies and small-molecule antagonists did not share the same mechanism and site of action; therefore, their activity might be synergic or contrasting and no cross-resistance was observed [55]. Resistance to a monoclonal antibody was only observed *in vitro*, where mAb-adapted HIV strains developed several mutations in gp120, becoming resistant to the antibody block [58]. The mechanism for the development of resistance to 2D7 was unclear, but it was related to the strong selective pressure exerted on the hypervariable V3 loop of HIV, that competed with 2D7 antibody for ECL2 binding. Despite CCR5 blocking, tested HIV strains did not show any R5-to-X4 shift in coreceptor usage. Results from this study led to formulate the hypothesis that antibodies recognizing multidomain, conformational epitopes, such as PA14/PRO140, should not induce resistance, due to the nature of their molecular targets and to their mechanism of action, that did not affect CCR5-gp120 interaction but post-binding events [59]. **Table **1 summarizes main biochemical and biological properties of antibodies described in the review.

**Table 1:** Antibodies recognizing CCR5 domains and their biologic properties

**Table 1.** Biological properties of some monoclonal and natural antibodies to CCR5; modified from [36].					
***Antibody***	***Source***	***Binding domain***	***Epitope(s)***	***Biologic properties***	***References***

2D7	mAb	ECL2	Q170, K171, E172, W190	Inhibition of chemokine binding Inhibition of cell activation (no Ca^++^ flux) R5-HIV blocking	[27]
PA9	mAb	N-term ECL2	D2, Y3, Q4, S7, P8, N13 Y176, T177	Inhibition of chemokine binding	[27]
PA14 PRO140	mAb hu mAb	N-term ECL2	D2 R168, Y176	Inhibition of chemokine binding Inhibition of cell activation (no Ca^++^ flux) R5-HIV blocking	[27] [35,56]
HGS004 (HGS101)	hu mAb	ECL2	Not available	Inhibition of chemokine binding without signaling HIV blocking	[57]
MC-1	mAb	ECL2	Not available	Inhibition of CCL4/MIP-1beta and CCL5/RANTES binding CCR5 dimerization CCR5 internalization Inhibition of R5-HIV binding	[26]
MC-4	mAb	ECL2	Not available	CCL5/RANTES-mediated signaling Inhibition of CCR5 endocytosis	[26]
MC-6	mAb	Multi-domain	conformational, multi-domain epitope including K171, E172	CCL5/RANTES signaling without CCR5 internalization	[26]
RoAb12 RoAb14 RoAb18	mAb	ECL2	K171, E172, W190	Inhibition of CCR5-mediated cell fusion Inhibition of CCL3/MIP-1alpha, CCL4/MIP-1beta, CCL5/RANTES binding Inhibition of cell activation (no Ca^++^ flux) Block of R5-HIV strains	[38,40]
Natural anti-CCR5 Abs	Healthy donors Delta32^+/+^ CCR5- ESNHIV-positive	ECL2 N-term	Unknown within R168-K197 sequence	Competition for chemokine binding Binding to native CCR5 on PBMC Block of R5-HIV laboratory and primary isolates	[1,15,16,21]
Natur alanti-CCR5 Abs	ESN LTNP	ECL1	D95, F96 A95, A96 (gain of function) A89, A103 (loss of function)	Inhibition of CCL4/MIP-1beta chemotaxis Binding to native CCR5 on PBMC CCR5 downregulation Block of HIV transcytosis across membranes Block of R5-HIV isolates from A, B, C, E clades	[22,29,30,33,81]

## Induction of anti-CCR5 immunity

Other experiments, carried out on mice and monkeys, showed that anti-CCR5 antibodies could be elicited in rodent, bird and monkey models and re-boosted when required [34,60,61]. Most importantly, anti-CCR5 IgG and IgA generated by immunization shared HIV-blocking properties with human monoclonal immunoglobulins and with natural antibodies found in exposed individuals [22,34].

Immunization experiments and *in vitro* studies of elicited antibodies were performed by Chain *et al*. [62], who immunized rabbits with chimeric peptides corresponding to a very short fragment of the N-terminal sequence of CCR5 (M1-S7 or D2-S7) and with a T-specific peptide from *Tetanus* toxoid. T-specific CCR5 epitopes were not included in the immunogen to prevent the development of host autoimmune responses. Immunization generated a strong antibody response; binding experiments to N-terminal and full-length CCR5 suggested that CCR5-binding antibodies were a small percentage of the total antibodies elicited by immunization; nevertheless, anti-CCR5 specific antibodies blocked HIV infection of macrophages *in vitro*. Devito *et al*. [63] carried out a long-term immunization with an intranasal DNA prime followed by a peptide booster immunization. Delivered antigens were peptides from gp120 V3 loop, from gp41 (MPER peptides containing the ELDKWAS epitope) and from the CCR5-ECL2 domain (R168–S185). The vaccination schedule elicited specific IgG and IgA in sera and in mucosal secretions (intestinal, vaginal and lung) in immunized mice. More interestingly, long-term IgG and IgA responses were still observed after 12 months from boosting both in serum and in mucosal secretions and still displayed HIV–blocking properties. According to this study, an intranasal DNA prime followed by one peptide/L3 adjuvant booster immunization, but not vice versa, induced long-lasting HIV-blocking antibodies and B memory cells to poorly immunogenic, conformational epitopes. Barassi *et al*. [34] generated chimeric immunogens containing a CCR5 peptide from the ECL1 domain (Y89–W102) in the context of the capsid protein of flock house virus, a conformation-constrained expression system [64]. Administered to mice by systemic or mucosal route, the immunogens elicited anti-CCR5 IgG and IgA both in sera and in vaginal fluids. Similarly to HIV-exposed seronegative individuals, mice producing anti-CCR5 autoantibodies expressed significantly reduced levels of CCR5 on the surfaces of CD4^+^ cells from peripheral blood and vaginal washes. *In vitro* studies showed that murine IgG and IgA (i) specifically bound human and mouse CD4^+^ lymphocytes and the CCR5-transfected U87 cell line; (ii) downregulated CCR5 expression of CD4^+^ cells from both humans and untreated mice; (iii) inhibited CCL4/MIP-1β chemotaxis of CD4^+^ CCR5^+^ lymphocytes and (iv) blocked *in vitro* infectivity of R5-HIV strains belonging to clade B. Finally, Pastori *et al*. [65] performed a peptide-scanning assay on a panel of synthetic peptides spanning the CCR5-ECL1 region; the resulting peptides were assayed with a pool of natural anti-CCR5 antibodies and used to immunize mice and chickens. Further structural characterization of the peptides was provided by NMR spectroscopy and by molecular dynamics simulations. Aminoacid substitutions in positions 95 and 96 (ECL1, A95–A96) increased antibody–peptide binding compared to the wild-type peptide (ECL1, D95–F96). The A95–A96 peptide was shown to induce, in both mice and chickens, antibodies displaying biological activity at very low concentrations. Strikingly, chicken antibodies to the modified peptide A95-A96 specifically recognized human CCR5 molecules, downregulated receptors from lymphocytes, inhibited CCR5-dependent chemotaxis and prevented infection by several R5 primary isolates belonging to Clades A, B, C and E, displaying IC_50_ values lower than 3 ng/ml. NMR spectroscopy and molecular dynamics simulations confirmed the high flexibility of the isolated epitopes and suggested that A95–A96 substitutions conferred a slightly higher tendency to generate helical conformations combined with a lower steric hindrance of the side chains in the peptides. The different structural behavior of the mutagenized loop might account for a better molecular structural organization, allowing the induction of the fittest antibodies. Optimized antibodies recognized and bound native CCR5 with higher affinity and displayed enhanced biological activity.

Other *in vivo* studies coupled immunization experiments with *in vivo* challenges of vaccinated animals to evaluate whether a break in B-tolerance was achieved and what was the extent of immune protection conferred by tested immunogens. Chackerian *et al*. [60] used the N-terminal domain of pigtailed macaque CCR5 fused to streptavidin. Once conjugated at high densities to the capsid protein L1 within bovine papilloma virus-like particles, this immunogen induced high-titer anti-CCR5 IgG that blocked infection by R5-tropic simian-human immunodeficiency virus (SHIV) *in vitro*. FACS analysis of spleen cells, thymus cells and PBMC did not detect any decline in the number of CCR5-expressing cells (T lymphocytes and macrophages) in immunized animals vs controls. In SHIV-challenged macaques, viral loads and time to control of viremia were significantly decreased in respect to controls, indicating that CCR5 auto-antibodies could have contributed to the control of viral replication. Bogers *et al*. [66] assayed a vaccine consisting of three extracellular peptides of CCR5, an N-terminal HIV gp120 fragment generated in transgenic plants and the recombinant simian immunodeficiency virus p27 antigen. They were linked to the microbial heat-shock protein HSP70, used as a carrier and the vaccine was administered by mucosal and systemic routes. Vaginal challenge with SHIV infected all macaques, with a significant variation in viral loads between immunized and control animals; the virus was cleared in five out of nine immunized animals. Misumi *et al*. [67] adopted synthetic cyclic peptides from the ECL2 (R168-T177) to induce anti-CCR5 antibodies in cynomolgus macaques. The immunization with a conjugated multiple-antigen peptide (cyclic closed chain dodecapeptide, cDDR5-MAP) induced long-lasting anti-cDDR5 antibodies reacting with both human and macaque CCR5 molecules, which suppressed *in vitro* infections by an R5-HIV-1 laboratory isolate, by R5-HIV-1 primary isolates belonging to clade A and C and by a pathogenic SHIV isolate. After SHIV challenge, the vaccinated cynomolgus macaques showed an attenuated acute infection and a lower viral load than the unvaccinated control animals.

According to *in vitro* and *in vivo* findings, immunization did elicit antibodies endowed with HIV-blocking properties, effectively breaking B-tolerance. Despite the fact that none of the immunogens assayed *in vivo* was able to confer full protection from virus challenge, the infection of vaccinated subjects was milder than in the controls and virus control was achieved in most subjects. Finally, *in vitro* studies also showed that conformational changes in the CCR5 protein, together with host factors, had the potential to modulate protein immunogenicity *in vivo* and might also play a role in the natural resistance to HIV infection.

## Conclusions

Natural antibodies offer a prompt and effective protection to most microbial infections and are likely to play a protective role in HIV infection as well. CCR5 is a key player in HIV entry and many medical approaches have been focused on it to prevent HIV infection and/or spread. The clinical use of small CCR5 inhibitors has proven the feasibility and the efficacy of CCR5 targeting, but it has also raised concerns about the safety of this approach: drug-resistant R5-HIV strains have been isolated in cell cultures and in patients receiving maraviroc and other CCR5 inhibitors [68-70]. The use of humanized monoclonal antibodies has proven effective and safe in HIV-infected patients, suggesting that passive immunization may offer therapeutic advantages [56,57]. The use of engineered chemokines induced receptor downregulation, removing CCR5 from availability for HIV binding; despite its effectiveness, this approach might be associated *in vivo* with adverse inflammatory events [71]. An HIV vaccine remains the most expected goal to be accomplished in HIV research, showing its value both in therapeutic intervention and in prevention [72]. Vaccination may offer long-lasting protection with few administrations, in a way acceptable in many geographical and social contexts, where other forms of prevention for sexually transmitted diseases could be impractical or rejected [73].

Anti-CCR5 vaccination is an innovative anti-HIV strategy, which could provide effective protection or safe containment to virus spread. Most importantly, anti-CCR5 antibodies raised in animal models or naturally occurring upon HIV exposure showed blocking activity to different virus clades, a result that was hardly achieved by conventional HIV-based immunogens [30,34,60]. Indeed, the feasibility of anti-CCR5 vaccination has been already demonstrated by two groups of naturally CCR5-deficient people. Individuals deprived of CCR5 receptor by genetic deletion [74-76] and those carrying naturally occurring anti-CCR5 antibodies downregulating the receptor *in vivo *[22,29,30] were found to be healthy and largely resistant to HIV-infection. Importantly, natural anti-CCR5 antibodies to the ECL1 domain have been uniquely observed in the sera and in mucosal fluids of individuals who remained uninfected despite repeated and unprotected sexual exposure to HIV and in HIV-infected individuals with long-term, asymptomatic infection. The finding that both ESN and LTNP subpopulations exerted a high and durable control on the virus confirmed the hypothesis that natural anti-CCR5 antibodies could be associated with protection. This concept was further strengthened by the good health and immune status shown by the LTNP cohort, confirming that long-lasting CCR5 downregulation was not harmful; conversely, cohort follow-up showed that the loss of anti-CCR5 responses experienced by some patients was associated with a decline in virus control [30]. These findings are noteworthy because genetic CCR5 deletion has been associated with an increased susceptibility to some viral and bacterial pathogens [77]; moreover, anti-self immunity was one of the mechanisms evoked to explain the generation of natural anti-CCR5 antibodies [53] and a possible adverse event associated with anti-CCR5 vaccination [55]. Conversely, CCR5 targeting could offer therapeutic advantages in some autoimmune diseases, as rheumatoid arthritis [78], or in transplantation therapy, all situations where chemokine signaling and cell recruitment might sustain tissue damage [79]. Another key finding from the follow-up of the LTNP cohort was the lack of an R5-to-X4 tropism shift, a fact supporting the safety of antibody-mediated coreceptor targeting [30]; this is a key point to be considered, due to the concerns raised by the therapeutic use of small-molecule CCR5 inhibitors, which are prone to *in vitro* and *in vivo* drug resistance and might favor the selection of dual-tropic or X4-tropic virus strains [54,68,80]. Indeed, immunization experiments performed in animal models have shown that anti-CCR5 antibodies can be obtained *in vivo*, provided that suitable vector systems are used, either to break B-tolerance to the self-CCR5 antigen and to constrain the ECL1 peptide (i.e. the target domain of these natural anti-CCR5 antibodies) in a conformation similar to the naturally occurring, immunogenic one [34,65]. Moreover, anti-CCR5 antibodies elicited by the mucosal route are long-lasting and can be promptly re-boosted upon immunization, either in sera or, most importantly, in mucosal fluids, showing the feasibility of local immunity at major portals of HIV entry [34].

This latter issue can sound paradoxical, because natural responses generated by B-1 cells should be independent from T-helper lymphocytes [2,7,10] and therefore could not give rise to memory cells, although some “memory” B-1 cells have been described [12,13]. However, the use of peculiar vectors and adjuvants in immunization experiments aimed at reproducing anti-CCR5 antibodies did induce a break in immune tolerance, and therefore could have achieved a forced, “non-natural” response to a self antigen; on the other hand, even the mechanisms leading to generate some types of anti-CCR5 antibodies in HIV-exposed people are still largely undefined [43,53].

Taken together, all of the findings reviewed here support the significance of interventions focused on CCR5 in its role as principal HIV coreceptor. Among all strategies now available or under development, naturally occurring anti-CCR5 antibodies show the therapeutic potential to provide durable, effective and safe systemic and, especially, local immunity to HIV. As shown by follow-up studies and immunization experiments, antibody-mediated CCR5 targeting was not only feasible but it was also well tolerated. Together with other immune-modulating strategies, this unconventional approach could open unprecedented avenues of treatment not only for HIV/AIDS but also for other disorders where harmful pro-inflammatory responses can develop.

## Competing interests

The author does not have any competing interest to declare.
